# 2,4-Bis(4-eth­oxy­phen­yl)-1-methyl-3-aza­bicyclo­[3.3.1]nonan-9-one

**DOI:** 10.1107/S1600536812039840

**Published:** 2012-09-26

**Authors:** Dong Ho Park, V. Ramkumar, P. Parthiban

**Affiliations:** a Department of Biomedicinal Chemistry, Inje University, Gimhae, Gyeongnam 621 749, Republic of Korea; b Department of Chemistry, IIT Madras, Chennai 600 036, TamilNadu, India

## Abstract

In the title compound, C_25_H_30_NO_3_, a crystallographic mirror plane bis­ects the mol­ecule. Although it is a positional isomer of 2,4-bis(4-eth­oxy­phen­yl)-7-methyl-3-aza­bicyclo­[3.3.1]non­an-9-one [C_25_H_31_NO_3_, *M* *_r_* = 393.51; Park *et al.* (2012*c* ▶). *Acta Cryst.* E**68**, o779–780], its mol­ecular weight is 392.50 due to the 50:50 ratio of the methyl group at bridgehead C atoms. However, the title compound exists in the same twin-chair conformation as its 7-methyl isomer. Also, the 4-eth­oxy­phenyl groups are equatorially oriented on the bicycle as in its isomer. In the title compound, the cyclo­hexanone ring deviates from an ideal chair (total puckering amplitude *Q*_T_ = 0.5390 Å) and the piperidone ring is closer to an ideal chair (*Q*_T_ = 0.6064 Å). These *Q*_T_ values are very similar to those of its isomer. Even though a center of symmetry passes through the 7-methyl analog, the benzene rings are oriented 26.11 (3)° with respect to each other, whereas the orientation is 53.10 (3)° for the title compound. The title compound exhibits inter­molecular N—H⋯O inter­actions [H⋯*A* = 2.25 (2) Å, *versus* 2.26 (2) Å for the analog].

## Related literature

For the synthesis, stereochemistry and biological activities of 3-aza­bicyclo­[3.3.1]nonan-9-ones, see: Park *et al.* (2011*a* ▶, 2012*a* ▶). For analogous structures, see: Park *et al.* (2012*b* ▶, 2012*c* ▶); Parthiban *et al.* (2011*b* ▶, 2011*c* ▶). For ring puckering parameters, see: Cremer & Pople (1975 ▶).
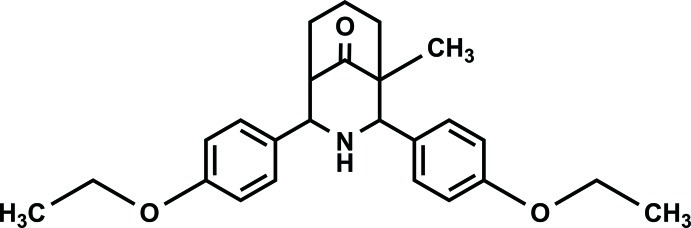


## Experimental

### 

#### Crystal data

C_25_H_30_NO_3_
*M* *_r_* = 392.50
Orthorhombic, 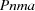

*a* = 11.9280 (4) Å
*b* = 26.1702 (14) Å
*c* = 6.9656 (3) Å
*V* = 2174.37 (17) Å^3^
*Z* = 4
Mo *K*α radiation
μ = 0.08 mm^−1^
*T* = 298 K
0.35 × 0.28 × 0.15 mm

#### Data collection

Bruker APEXII CCD area-detector diffractometer
Absorption correction: multi-scan (*SADABS*; Bruker, 2004 ▶) *T*_min_ = 0.973, *T*_max_ = 0.988
7809 measured reflections
2396 independent reflections
1689 reflections with *I* > 2σ(*I*)
*R*_int_ = 0.029

#### Refinement

*R*[*F* ^2^ > 2σ(*F* ^2^)] = 0.047
*wR*(*F* ^2^) = 0.133
*S* = 1.06
2396 reflections
147 parameters
H atoms treated by a mixture of independent and constrained refinement
Δρ_max_ = 0.22 e Å^−3^
Δρ_min_ = −0.17 e Å^−3^

### 

Data collection: *APEX2* (Bruker, 2004 ▶); cell refinement: *APEX2* and *SAINT-Plus* (Bruker, 2004 ▶); data reduction: *SAINT-Plus* and *XPREP* (Bruker, 2004 ▶); program(s) used to solve structure: *SHELXS97* (Sheldrick, 2008 ▶); program(s) used to refine structure: *SHELXL97* (Sheldrick, 2008 ▶); molecular graphics: *ORTEP-3* (Farrugia, 1997 ▶); software used to prepare material for publication: *SHELXL97*.

## Supplementary Material

Crystal structure: contains datablock(s) global, I. DOI: 10.1107/S1600536812039840/bq2375sup1.cif

Structure factors: contains datablock(s) I. DOI: 10.1107/S1600536812039840/bq2375Isup2.hkl

Supplementary material file. DOI: 10.1107/S1600536812039840/bq2375Isup3.cml

Additional supplementary materials:  crystallographic information; 3D view; checkCIF report

## Figures and Tables

**Table 1:** Hydrogen-bond geometry (Å, °)

*D*—H⋯*A*	*D*—H	H⋯*A*	*D*⋯*A*	*D*—H⋯*A*
N1—H1*N*⋯O1^i^	0.86 (2)	2.25 (2)	3.105 (2)	180 (2)

Symmetry code: (i) 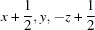.

**Fig. 1.**
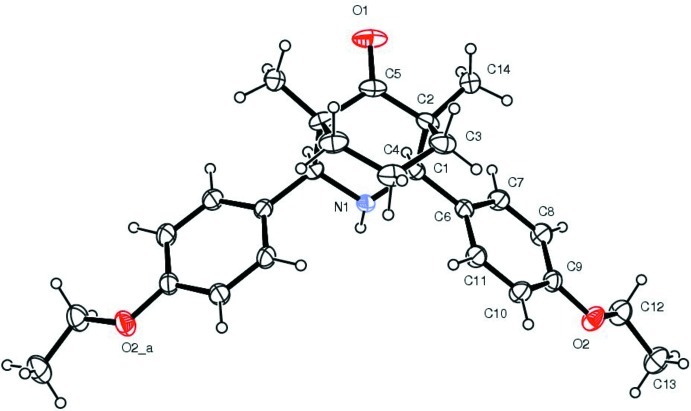
Anisotropic displacement representation of the molecule with atoms represented with 30% probability ellipsoids.

**Fig. 2.**
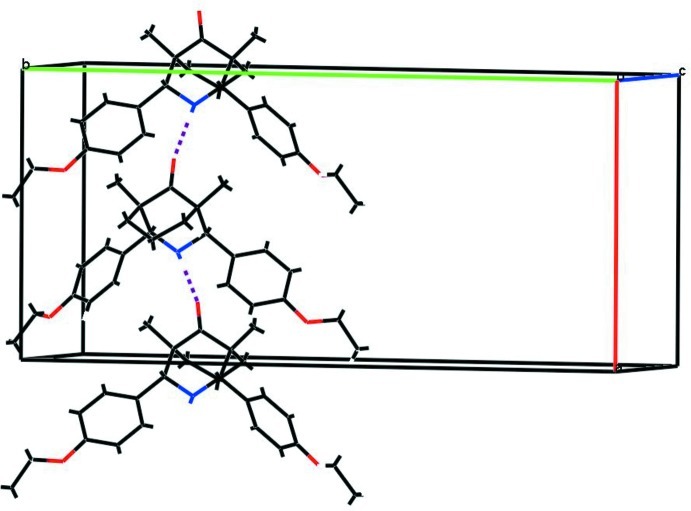
Intermolecular N—H···O interactions present in the molecule.

## supplementary crystallographic information

### Comment

Synthesis and stereochemical analysis of the 3-azabicyclononanes are interesting due to their biological actions (Park *et al.*, 2012*a*). Stereochemical analysis of the biologically active molecules are crucial in drug-design and drug-development programs. Hence, we synthesized the title compound by a modified and an optimized successive double Mannich condensation in one-pot in order to explore its stereochemistry in solid-state.

The detailed analysis and comparison of the torsion angles clearly indicates that the title molecule, C_25_H_30_NO_3_, exists in a twin-chair conformation with an equatorial orientation of the 4-ethoxyphenyl groups on both sides of the secondary amino group.

Further, the Cremer & Pople (Cremer & Pople, 1975) ring puckering parameters calculated for the title compound shows that the piperidone ring adopts a near ideal chair conformation [The total puckering amplitude, Q_T_ is 0.6064 Å, the phase angle θ is 175.10° and phi is 359.99°], and the cyclohexane exist in a distorted chair conformation [Q_T_ = 0.5390, θ = 9.52° and phi = 60.00°]

The crystal packing of the title compound, 2,4-Bis(4-ethoxyphenyl)-1-methyl-3-azabicyclo[3.3.1]nonan-9-one is stabilized by intermolecular N—H···O interaction (Table 1) as its 7-methyl analog of 2.26 (2) Å (Park *et al.*, 2012*c*).

### Experimental

The title compound was synthesized by a modified and an optimized Mannich condensation in one-pot, using 4-ethoxybenzaldehyde (0.1 mol, 15.018 g/13.91 ml), 2-methylcyclohexanone (0.05 mol, 5.61 g/6.07 ml) and ammonium acetate (0.075 mol, 5.78 g) in a 50 ml of absolute ethanol (Park *et al.*, 2011*a*). The mixture was gently warmed on a hot plate at 303–308 K (30–35° C) with moderate stirring till the complete consumption of the starting materials, which was monitored by TLC. At the end, the crude azabicyclic ketone was separated by filtration and gently washed with 1:5 cold ethanol-ether mixture. X-ray diffraction quality crystals of the title compound were obtained by slow evaporation from ethanol.

### Refinement

The nitrogen H atom was located in a difference Fourier map and refined isotropically. Other hydrogen atoms were fixed geometrically and allowed to ride on the parent carbon atoms with aromatic C—H = 0.93 Å, aliphatic C—H = 0.98 Å and methylene C—H = 0.97 Å. The displacement parameters were set for phenyl, methylene and aliphatic H atoms at *U*_iso_(H) = 1.2*U*_eq_(C) and for methyl H atoms at *U*_iso_(H) = 1.5*U*_eq_(C).

### Crystal data

**Table:**

C_25_H_30_NO_3_	*F*(000) = 844
*M**_r_* = 392.50	*D*_x_ = 1.199 Mg m^−^^3^
Orthorhombic, *P**n**m**a*	Mo *K*α radiation, λ = 0.71073 Å
Hall symbol: -P 2ac 2n	Cell parameters from 3421 reflections
*a* = 11.9280 (4) Å	θ = 3.0–27.7°
*b* = 26.1702 (14) Å	µ = 0.08 mm^−^^1^
*c* = 6.9656 (3) Å	*T* = 298 K
*V* = 2174.37 (17) Å^3^	Block, colourless
*Z* = 4	0.35 × 0.28 × 0.15 mm

### Data collection

**Table:**

Bruker APEXII CCD area-detector diffractometer	2396 independent reflections
Radiation source: fine-focus sealed tube	1689 reflections with *I* > 2σ(*I*)
Graphite monochromator	*R*_int_ = 0.029
phi and ω scans	θ_max_ = 28.4°, θ_min_ = 3.0°
Absorption correction: multi-scan (*SADABS*; Bruker, 2004)	*h* = −15→14
*T*_min_ = 0.973, *T*_max_ = 0.988	*k* = −33→25
7809 measured reflections	*l* = −8→8

### Refinement

**Table:**

Refinement on *F*^2^	Primary atom site location: structure-invariant direct methods
Least-squares matrix: full	Secondary atom site location: difference Fourier map
*R*[*F*^2^ > 2σ(*F*^2^)] = 0.047	Hydrogen site location: inferred from neighbouring sites
*wR*(*F*^2^) = 0.133	H atoms treated by a mixture of independent and constrained refinement
*S* = 1.06	*w* = 1/[σ^2^(*F*_o_^2^) + (0.0613*P*)^2^ + 0.3421*P*] where *P* = (*F*_o_^2^ + 2*F*_c_^2^)/3
2396 reflections	(Δ/σ)_max_ < 0.001
147 parameters	Δρ_max_ = 0.22 e Å^−^^3^
0 restraints	Δρ_min_ = −0.17 e Å^−^^3^

### Special details

**Table:**

Geometry. All e.s.d.'s (except the e.s.d. in the dihedral angle between two l.s. planes)are estimated using the full covariance matrix. The cell e.s.d.'s are takeninto account individually in the estimation of e.s.d.'s in distances, anglesand torsion angles; correlations between e.s.d.'s in cell parameters are onlyused when they are defined by crystal symmetry. An approximate (isotropic)treatment of cell e.s.d.'s is used for estimating e.s.d.'s involving l.s. planes.
Refinement. Refinement of *F*^2^ against ALL reflections. The weighted *R*-factor *wR* and goodness of fit *S* are based on *F*^2^, conventional *R*-factors *R* are based on *F*, with *F* set to zero for negative *F*^2^. The threshold expression of *F*^2^ > σ(*F*^2^) is used only for calculating *R*-factors(gt) *etc*. and is not relevant to the choice of reflections for refinement. *R*-factors based on *F*^2^ are statistically about twice as large as those based on *F*, and *R*- factors based on ALL data will be even larger.

### Fractional atomic coordinates and isotropic or equivalent isotropic displacement parameters (Å^2^)

**Table:**

	*x*	*y*	*z*	*U*_iso_*/*U*_eq_	Occ. (<1)
C1	0.55634 (11)	0.70312 (5)	0.13583 (19)	0.0350 (4)	
H1	0.5180	0.7037	0.2603	0.042*	
C2	0.46403 (11)	0.70192 (6)	−0.0240 (2)	0.0419 (4)	
C3	0.39793 (16)	0.7500	0.0051 (3)	0.0444 (6)	
C4	0.57166 (18)	0.7500	−0.2899 (3)	0.0487 (6)	
H4A	0.5826	0.7500	−0.4279	0.058*	
H4B	0.6450	0.7500	−0.2296	0.058*	
C5	0.50932 (12)	0.70215 (7)	−0.2323 (2)	0.0511 (5)	
H5A	0.5591	0.6731	−0.2482	0.061*	
H5B	0.4467	0.6975	−0.3195	0.061*	
C6	0.63304 (11)	0.65729 (5)	0.1344 (2)	0.0360 (4)	
C7	0.72669 (13)	0.65458 (6)	0.0151 (2)	0.0445 (4)	
H7	0.7415	0.6814	−0.0687	0.053*	
C8	0.79747 (13)	0.61325 (6)	0.0183 (2)	0.0464 (4)	
H8	0.8589	0.6123	−0.0638	0.056*	
C9	0.77820 (12)	0.57292 (6)	0.1430 (2)	0.0415 (4)	
C10	0.68483 (13)	0.57420 (6)	0.2607 (2)	0.0468 (4)	
H10	0.6697	0.5471	0.3433	0.056*	
C11	0.61410 (12)	0.61616 (6)	0.2545 (2)	0.0440 (4)	
H11	0.5515	0.6167	0.3341	0.053*	
C12	0.84722 (15)	0.49538 (6)	0.2799 (2)	0.0564 (5)	
H12A	0.7787	0.4760	0.2626	0.068*	
H12B	0.8457	0.5107	0.4066	0.068*	
C13	0.94628 (17)	0.46091 (7)	0.2622 (3)	0.0651 (5)	
H13A	0.9492	0.4471	0.1347	0.098*	
H13B	0.9398	0.4335	0.3533	0.098*	
H13C	1.0135	0.4799	0.2873	0.098*	
C14	0.3844 (2)	0.66095 (12)	−0.0007 (4)	0.0431 (7)	0.50
H14A	0.3580	0.6604	0.1295	0.065*	0.50
H14B	0.4199	0.6290	−0.0301	0.065*	0.50
H14C	0.3222	0.6662	−0.0860	0.065*	0.50
N1	0.62172 (13)	0.7500	0.1205 (2)	0.0327 (4)	
O1	0.29964 (12)	0.7500	0.0541 (3)	0.0738 (6)	
O2	0.85487 (9)	0.53399 (4)	0.13792 (16)	0.0533 (3)	
H1N	0.6708 (19)	0.7500	0.211 (3)	0.039 (6)*	

### Atomic displacement parameters (Å^2^)

**Table:**

	*U*^11^	*U*^22^	*U*^33^	*U*^12^	*U*^13^	*U*^23^
C1	0.0331 (6)	0.0380 (9)	0.0340 (8)	−0.0042 (7)	0.0035 (5)	0.0000 (6)
C2	0.0309 (7)	0.0523 (11)	0.0425 (8)	−0.0109 (7)	−0.0005 (6)	−0.0055 (7)
C3	0.0272 (9)	0.0715 (17)	0.0343 (11)	0.000	−0.0034 (7)	0.000
C4	0.0385 (10)	0.0767 (17)	0.0310 (11)	0.000	0.0012 (8)	0.000
C5	0.0407 (7)	0.0714 (12)	0.0411 (9)	−0.0051 (9)	−0.0045 (6)	−0.0121 (8)
C6	0.0358 (7)	0.0327 (9)	0.0394 (8)	−0.0046 (6)	0.0022 (6)	0.0006 (6)
C7	0.0460 (8)	0.0342 (9)	0.0534 (9)	−0.0021 (7)	0.0130 (7)	0.0109 (7)
C8	0.0432 (8)	0.0413 (10)	0.0545 (9)	0.0028 (8)	0.0149 (7)	0.0096 (7)
C9	0.0458 (8)	0.0326 (8)	0.0462 (9)	0.0019 (7)	0.0037 (6)	0.0032 (7)
C10	0.0517 (9)	0.0389 (9)	0.0498 (9)	−0.0017 (8)	0.0087 (7)	0.0134 (7)
C11	0.0420 (8)	0.0440 (10)	0.0459 (9)	−0.0016 (7)	0.0109 (6)	0.0079 (7)
C12	0.0664 (11)	0.0465 (11)	0.0563 (10)	0.0091 (9)	0.0037 (8)	0.0147 (8)
C13	0.0728 (12)	0.0554 (12)	0.0672 (12)	0.0188 (10)	−0.0016 (9)	0.0133 (9)
C14	0.0435 (15)	0.0369 (18)	0.0488 (17)	−0.0060 (14)	0.0005 (13)	−0.0014 (14)
N1	0.0284 (8)	0.0319 (10)	0.0379 (9)	0.000	−0.0063 (7)	0.000
O1	0.0278 (7)	0.1163 (17)	0.0774 (12)	0.000	0.0094 (7)	0.000
O2	0.0591 (7)	0.0415 (7)	0.0594 (7)	0.0129 (6)	0.0127 (5)	0.0146 (5)

### Geometric parameters (Å, º)

**Table:**

C1—N1	1.4577 (16)	C8—C9	1.386 (2)
C1—C6	1.5084 (19)	C8—H8	0.9300
C1—C2	1.5661 (19)	C9—O2	1.3696 (18)
C1—H1	0.9800	C9—C10	1.383 (2)
C2—C14	1.442 (3)	C10—C11	1.385 (2)
C2—C3	1.4986 (19)	C10—H10	0.9300
C2—C5	1.549 (2)	C11—H11	0.9300
C3—O1	1.221 (2)	C12—O2	1.4169 (18)
C3—C2^i^	1.4986 (19)	C12—C13	1.492 (2)
C4—C5	1.511 (2)	C12—H12A	0.9700
C4—C5^i^	1.511 (2)	C12—H12B	0.9700
C4—H4A	0.9700	C13—H13A	0.9600
C4—H4B	0.9700	C13—H13B	0.9600
C5—H5A	0.9700	C13—H13C	0.9600
C5—H5B	0.9700	C14—H14A	0.9600
C6—C11	1.3817 (19)	C14—H14B	0.9600
C6—C7	1.394 (2)	C14—H14C	0.9600
C7—C8	1.372 (2)	N1—C1^i^	1.4577 (16)
C7—H7	0.9300	N1—H1N	0.86 (2)
			
N1—C1—C6	110.13 (11)	C7—C8—H8	119.7
N1—C1—C2	109.94 (12)	C9—C8—H8	119.7
C6—C1—C2	113.94 (11)	O2—C9—C10	124.82 (13)
N1—C1—H1	107.5	O2—C9—C8	116.07 (12)
C6—C1—H1	107.5	C10—C9—C8	119.11 (14)
C2—C1—H1	107.5	C9—C10—C11	119.40 (14)
C14—C2—C3	105.22 (16)	C9—C10—H10	120.3
C14—C2—C5	109.76 (16)	C11—C10—H10	120.3
C3—C2—C5	107.89 (14)	C6—C11—C10	122.48 (13)
C14—C2—C1	113.46 (17)	C6—C11—H11	118.8
C3—C2—C1	104.89 (12)	C10—C11—H11	118.8
C5—C2—C1	114.89 (11)	O2—C12—C13	108.82 (14)
O1—C3—C2	122.89 (8)	O2—C12—H12A	109.9
O1—C3—C2^i^	122.89 (8)	C13—C12—H12A	109.9
C2—C3—C2^i^	114.19 (16)	O2—C12—H12B	109.9
C5—C4—C5^i^	112.00 (17)	C13—C12—H12B	109.9
C5—C4—H4A	109.2	H12A—C12—H12B	108.3
C5^i^—C4—H4A	109.2	C12—C13—H13A	109.5
C5—C4—H4B	109.2	C12—C13—H13B	109.5
C5^i^—C4—H4B	109.2	H13A—C13—H13B	109.5
H4A—C4—H4B	107.9	C12—C13—H13C	109.5
C4—C5—C2	115.05 (14)	H13A—C13—H13C	109.5
C4—C5—H5A	108.5	H13B—C13—H13C	109.5
C2—C5—H5A	108.5	C2—C14—H14A	109.5
C4—C5—H5B	108.5	C2—C14—H14B	109.5
C2—C5—H5B	108.5	H14A—C14—H14B	109.5
H5A—C5—H5B	107.5	C2—C14—H14C	109.5
C11—C6—C7	116.88 (13)	H14A—C14—H14C	109.5
C11—C6—C1	121.07 (12)	H14B—C14—H14C	109.5
C7—C6—C1	122.04 (12)	C1—N1—C1^i^	114.64 (14)
C8—C7—C6	121.55 (14)	C1—N1—H1N	108.1 (7)
C8—C7—H7	119.2	C1^i^—N1—H1N	108.1 (7)
C6—C7—H7	119.2	C9—O2—C12	117.99 (12)
C7—C8—C9	120.56 (13)		
			
N1—C1—C2—C14	−170.69 (17)	N1—C1—C6—C7	−39.74 (18)
C6—C1—C2—C14	65.14 (19)	C2—C1—C6—C7	84.32 (17)
N1—C1—C2—C3	−56.39 (15)	C11—C6—C7—C8	−0.7 (2)
C6—C1—C2—C3	179.44 (11)	C1—C6—C7—C8	178.36 (14)
N1—C1—C2—C5	61.89 (17)	C6—C7—C8—C9	−0.7 (2)
C6—C1—C2—C5	−62.28 (17)	C7—C8—C9—O2	−178.74 (14)
C14—C2—C3—O1	5.4 (3)	C7—C8—C9—C10	1.8 (2)
C5—C2—C3—O1	122.6 (2)	O2—C9—C10—C11	179.14 (14)
C1—C2—C3—O1	−114.5 (2)	C8—C9—C10—C11	−1.4 (2)
C14—C2—C3—C2^i^	−176.73 (16)	C7—C6—C11—C10	1.1 (2)
C5—C2—C3—C2^i^	−59.60 (19)	C1—C6—C11—C10	−178.02 (14)
C1—C2—C3—C2^i^	63.32 (19)	C9—C10—C11—C6	0.0 (2)
C5^i^—C4—C5—C2	−46.7 (2)	C6—C1—N1—C1^i^	−175.39 (10)
C14—C2—C5—C4	166.11 (18)	C2—C1—N1—C1^i^	58.26 (18)
C3—C2—C5—C4	51.97 (17)	C10—C9—O2—C12	−8.8 (2)
C1—C2—C5—C4	−64.62 (19)	C8—C9—O2—C12	171.71 (15)
N1—C1—C6—C11	139.30 (14)	C13—C12—O2—C9	−173.00 (14)
C2—C1—C6—C11	−96.64 (16)		

Symmetry code: (i) *x*, −*y*+3/2, *z*.

### Hydrogen-bond geometry (Å, º)

**Table:**

*D*—H···*A*	*D*—H	H···*A*	*D*···*A*	*D*—H···*A*
N1—H1*N*···O1^ii^	0.86 (2)	2.25 (2)	3.105 (2)	180 (2)

Symmetry code: (ii) *x*+1/2, *y*, −*z*+1/2.
